# Spatial Distribution Characteristics of Micronutrients and Their Deficiency Effect on the Root Morphology and Architecture in Citrus Rootstock

**DOI:** 10.3390/plants14020158

**Published:** 2025-01-08

**Authors:** Gaofeng Zhou, Yiping Fu, Mei Yang, Yanhong Li, Jing Zhang

**Affiliations:** 1 National Navel Orange Engineering Research Center, College of Navel Orange, Gannan Normal University, Ganzhou 341000, China; zhougaofeng428@163.com (G.Z.); fuyiping2024@163.com (Y.F.); yangmei208@163.com (M.Y.); liyanhong_208@163.com (Y.L.); 2 International Education School, Gannan Normal University, Ganzhou 341000, China

**Keywords:** citrus, lateral root, nutrient acquisition, nutrient deficiency, root architecture, root morphology

## Abstract

Roots play essential roles in the acquisition of water and minerals from soils in higher plants. However, water or nutrient limitation can alter plant root morphology. To clarify the spatial distribution characteristics of essential nutrients in citrus roots and the influence mechanism of micronutrient deficiency on citrus root morphology and architecture, especially the effects on lateral root (LR) growth and development, two commonly used citrus rootstocks, trifoliate orange (*Poncirus trifoliata* L. Raf., Ptr) and red tangerine (*Citrus reticulata* Blanco, Cre), were employed here. The analysis of the mineral nutrient distribution characteristics in different root parts showed that, except for the P concentrations in Ptr, the last two LR levels (second and third LRs) had the highest macronutrient concentrations. All micronutrient concentrations in the second and third LRs of Ptr were higher than those of Cre, except for the Zn concentration in the second LR, which indicates that Ptr requires more micronutrients to maintain normal root system growth and development. Principal component analysis (PCA) showed that B and P were very close in terms of spatial distribution and that Mo, Mn, Cu, and Fe contributed significantly to PC1, while B, Cu, Mo, and Zn contributed significantly to PC2 in both rootstocks. These results suggest that micronutrients are major factors in citrus root growth and development. The analysis of root morphology under micronutrient deficiency showed that root growth was more significantly inhibited in Ptr and Cre under Fe deficiency (FeD) than under other micronutrient deficiencies, while Cre roots exhibited better performance than Ptr roots. From the perspective of micronutrient deficiency, FeD and B deficiency (BD) inhibited all root morphological traits in Ptr and Cre except the average root diameter, while Mn deficiency (MnD) and Zn deficiency (ZnD) had lesser impacts, as well as the morphology of the stem. The mineral nutrient concentrations in Ptr and Cre seedlings under micronutrient deficiency revealed that single micronutrient deficiencies affected both their own concentrations and the concentrations of other mineral nutrients, whether in the roots or in stems and leaves. Dynamic analysis of LR development revealed that there were no significant decreases in either the first or second LR number in Ptr seedlings under BD and ZnD stress. Moreover, the growth rates of first and second LRs in Ptr and Cre did not significantly decrease compared with the control under short-term (10 days) BD stress. Altogether, these results indicate that micronutrients play essential roles in citrus root growth and development. Moreover, citrus alters its root morphology and biological traits as a nutrient acquisition strategy to maintain maximal micronutrient acquisition and growth. The present work on the spatial distribution characteristics and micronutrient deficiency of citrus roots provides a theoretical basis for effective micronutrient fertilization and the diagnosis of micronutrient deficiency in citrus.

## 1. Introduction

Root systems play many essential roles in adaptive functions in higher plants, including water and nutrient uptake, anchorage to the soil, and the establishment of biotic interactions in the rhizosphere [1]. Roots serve as the interface between plants and the complex soil environment. The acquisition of water and minerals from the soil is the key function of plant roots, and well-developed root system architecture (RSA) is essential for the realization of this function [2]. However, the morphological traits of plant roots can change when water or nutrients are limited. According to the latest reviews on the roles of plant root traits in acquiring mineral nutrients, root traits influence the acquisition of mineral nutrients by plants through six main processes: (1) high-affinity/high-capacity transport systems; (2) the modification of the rhizosphere pH and the efflux of low-molecular-weight organic solutes and/or enzymes from roots; (3) the distribution of roots in the soil profile; (4) the relative biomass allocation to the roots (root/shoot biomass quotient) and the root growth rate; (5) the architectural and anatomical characteristics of the root system; and (6) interactions with microorganisms [3]. Therefore, advantageous root traits play a fundamental role in improving the efficiency of water and nutrient acquisition.

Many previous studies have focused on the role of nutrient availability in regulating root architecture. The ability of plants to respond appropriately to nutrient availability is of fundamental importance for their adaptation to the environment. Deficiencies of nutrients such as nitrogen (N) and phosphorus (P) act as signals that can be perceived. These signals trigger molecular and physiological mechanisms that modify cell division and cell differentiation processes within the root and have a profound impact on RSA [1]. The relationship between P deficiency and plant RSA has been elucidated in numerous plants by many authors. Low P availability increases lateral root (LR) elongation and the number of LRs, whereas taproot root growth is reduced [4,5,6]. Under low P concentrations, mitotic activity is relocated to the sites of LR formation, which leads to increased LR density [7,8]. With respect to N, low N promotes LR growth but has no significant effect on taproot growth [9,10]. The molecular mechanisms that control taproot and LR development and growth under P and N deficiency have mainly been explored separately [11,12,13]. Comparatively few studies have focused on the effects of micronutrient deficiency on root development and growth.

Citrus is one of the most important fruit crops produced in tropical and subtropical regions around the world. However, citrus production can be seriously affected by micronutrient limitations, such as boron (B) [14], zinc (Zn) [15], and iron (Fe) limitations [16]. Grafting is widely employed in citrus production, and good citrus production, therefore, depends mainly on the availability of suitable rootstocks, which can influence citrus growth, yield, fruit quality, and tolerance to different biotic and abiotic stresses [17,18]. Previous research has shown that micronutrient shortages, which can also affect the RSA, have become one of the major factors hindering citrus production around the world. Over the last decade, most studies in this area have examined the effects of B deficiency on citrus root [19,20,21,22]. In contrast, very little research has been conducted on other micronutrients in citrus rootstocks, and there is little information regarding how citrus root traits improve micronutrient acquisition and the necessity of different traits under micronutrient limitations.

It is well known that mineral nutrients are essential for citrus plant growth and development, fruit yield, and quality. Therefore, effective fertilization is one of the most important management measures in citrus cultivation. Fertilization recommendations are proposed based on the mobility and distribution of mineral nutrients, and the mobility and distribution of the same mineral nutrients differ significantly in different crops [23]. RSA and root system function are important for crop yield and stress tolerance. The root system is the main organ involved in mineral nutrient absorption. Therefore, studying the distribution characteristics of mineral nutrients in the root system holds reference significance for nutrient management in citrus. As detailed in previous research, root nutrient acquisition includes proliferation, transporter function, exudation, symbioses, and the delivery of dissolved nutrients from the bulk soil to the root surface via mass flow and diffusion [24]. Hence, shaping a good citrus RSA is a key factor in improving root nutrient acquisition. In this work, to explore the effects of micronutrient deficiency on citrus RSA and the differences in root traits under different micronutrient deficiency conditions, two of the most commonly used citrus rootstocks, trifoliate orange (Ptr) and red tangerine (Cre), were used to conduct the subsequent experiments. The aims of this study were to (1) examine the mineral nutrient distribution in different parts of the root system (the taproot and three levels of LRs); (2) determine the effects of micronutrient Fe, manganese (Mn), B, and Zn deficiency on the root traits of the two rootstocks, including root growth, root morphology, and root nutrient accumulation; and (3) clarify the relationships between micronutrients and the RSA of citrus, especially in regard to the effects on LR growth and development. It is well known that improved root nutrient acquisition can enhance fertilizer use efficiency and is an important factor in securing food production. Therefore, research on the effects of micronutrients on the roots of citrus rootstocks is vital to increase micronutrient fertilizer use efficiency and improve citrus fruit product quality.

## 2. Results

### 2.1. Mineral Nutrient Distribution in Different Root Parts

**Figure 1:**
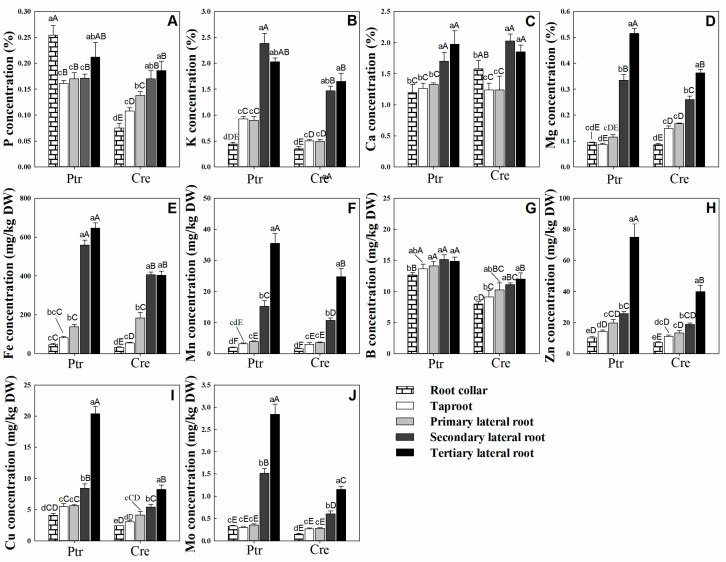
Nutrient distribution in different root parts of trifoliate orange (Ptr) and red tangerine (Cre) seedlings. (**A**) P concentration; (**B**) K concentration; (**C**) Ca concentration; (**D**) Mg concentration; (**E**) Fe concentration; (**F**) Mn concentration; (**G**) B concentration; (**H**) Zn concentration; (**I**) Cu concentration; (**J**) Mo concentration. Data are presented as the mean ± SE of six biological replicates. Different lowercase and uppercase letters above the bars indicate significant differences (*p* < 0.05) between the different root parts and the citrus rootstock species, respectively.

As shown in Figure 1A, the concentration of P was significantly higher in the root collar than in the taproot, but there was no significant difference between the concentrations of P in LRs at all levels. The K concentration in Ptr and Cre showed the same trend, with the second lateral root (second LR) and tertiary lateral root (third LR) having the highest K concentrations, significantly higher than those in the taproot and primary lateral root (first LR), while the root collar had the lowest K concentration (Figure 1B). For Ca, there was no significant difference between the Ca concentration of the taproot and that of the first LR, as well as between the second LR and third LR, but the former was significantly higher than the latter in both Ptr and Cre (Figure 1C). The Mg concentration decreased significantly in the order of third LR, second LR, and first LR in both Ptr and Cre (Figure 1D). Overall, except for the P concentrations in Ptr, the last two levels (second LR and third LR) had the highest concentrations of macronutrients.

Among the micronutrients, the Fe concentration was remarkably higher in both the second LR and third LR than in the other parts of the root system of Ptr (Figure 1E). This trend was also observed in Cre. The Fe concentration of the first LR was significantly higher than that of the root collar for both rootstocks. Similar trends were observed for Mn, Zn, Cu, and Mo, which exhibited significant increases in concentration in the order of root collar, taproot, first LR, second LR, and third LR in both Ptr and Cre (Figure 1F,H–J). For the B concentration in Ptr, while there were no significant differences between the B concentrations at all LR levels and the taproot, the B concentrations at all root levels were significantly higher than that in the root collar. In Cre, no significant differences were found between the B concentrations at all LR levels, but the taproot B concentration was significantly higher than that in the root collar and lower than the B concentrations of the second LR and third LR (Figure 1G). Taken as a whole, all micronutrient concentrations in the last two root levels (second LR and third LR) of Ptr were higher than those of Cre, except for the Zn concentration in the second LR. These results indicate that Ptr requires more micronutrients than Cre to maintain the normal growth and development of the root system.

### 2.2. Principal Component Analysis of Nutrient Distribution

**Figure 2:**
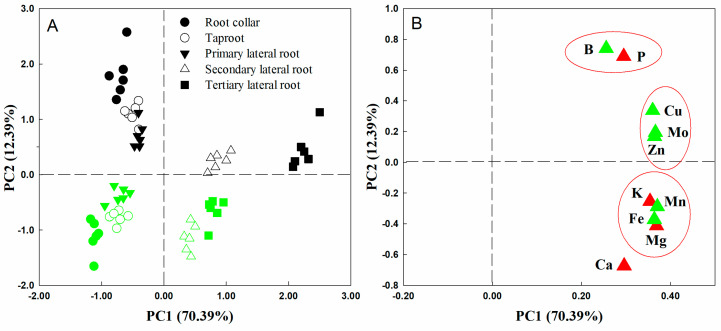
Principal component analysis (**A**) and loading scatter plot (**B**) of 10 elements in different root parts of trifoliate orange and red tangerine seedlings. Green symbols in (**A**) represent red tangerine, and black symbols represent trifoliate orange; green symbols in (**B**) represent micronutrients, and red symbols represent macronutrients. The red circle in subfigure (**B**) indicated that the correlation between nutrient elements was significant.

The principal component analysis (PCA) results showed that the first and second principal components could explain 70.39% and 12.39% of the total variance, respectively. The first component generally distinguished the different root parts, particularly the second and third LRs, while the second component separated the roots of Ptr from those of Cre (Figure 2A). As shown in Figure 2B, loading scatter plots of 10 elements in different root parts were analyzed. The results showed that Mo and Mn contributed more to PC1, while B, P, and Ca contributed more to PC2. Furthermore, B and P were very close in terms of spatial distribution. This result implies that P and B may play similar roles in root growth and development. Interestingly, no elements were distributed in the negative area of PC1.

### 2.3. Root Morphology Under Micronutrient Deficiency Conditions

**Figure 3:**
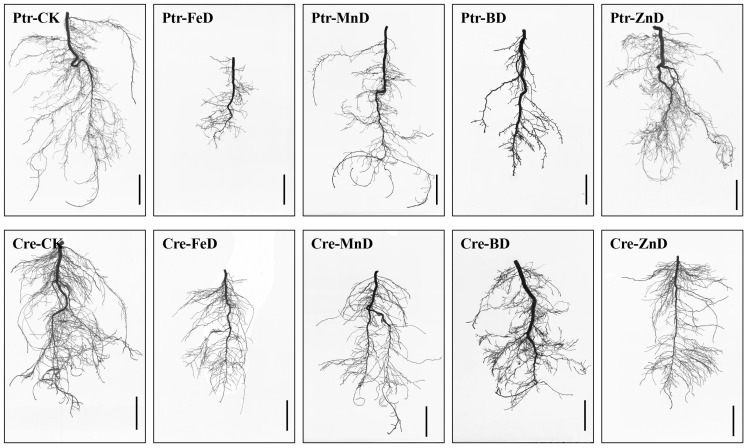
Scanned images of the root morphology of two types of citrus rootstocks under different micronutrient deficiency conditions. Ptr: trifoliate orange, Cre: red tangerine, CK: control, FeD: iron deficiency, MnD: manganese deficiency, BD: boron deficiency, and ZnD: zinc deficiency. Bar = 2 cm.

Visible symptoms and plant growth are depicted in Figure 3. Root growth was significantly inhibited in both Ptr and Cre under iron deficiency (FeD) conditions compared with other micronutrient deficiencies, but the Cre roots exhibited better performance than the Ptr roots under FeD. For manganese deficiency (MnD) treatments, the LRs number was obviously reduced and the taproot became thinner in diameter, but the reduction in taproot length was lower than that observed in other micronutrient deficiency treatments in both Ptr and Cre. In the zinc deficiency (ZnD) treatment, the root morphology was slightly affected compared with other treatments. There were no significant differences in root morphology between Ptr and Cre following the MnD and ZnD treatments. The most dramatic morphological difference was found between the roots of Ptr and Cre under the boron deficiency (BD) treatment. A large number of short second LRs were formed in the root system of Ptr under BD conditions, while this was not observed in the Cre root system. Effects of micronutrient deficiency stress on stem growth parameters in Ptr and Cre seedlings were also measured. Stem lengths (seedling height) were decreased significantly in both Ptr and Cre under micronutrient deficiency conditions, except for Ptr under ZnD. Stem diameter decreased significantly in Ptr under FeD and in Cre under FeD, MnD, and ZnD treatment but increased significantly in Ptr under BD. Stem and root dry weight were decreased significantly in Cre under all micronutrient deficiency treatments. For Ptr, the stem dry weights decreased significantly under FeD and MnD but increased significantly under BD and ZnD conditions (Table 1).

### 2.4. RSA Assay

**Figure 4:**
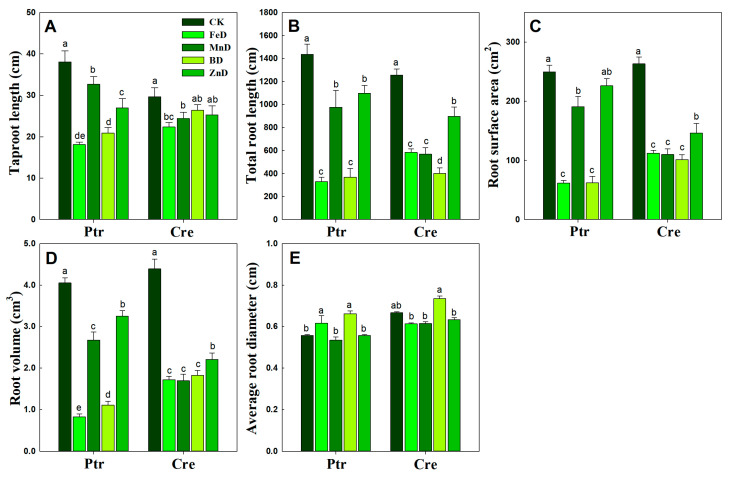
Root system architecture response to micronutrient deficiency stress in trifoliate orange (Ptr) and red tangerine (Cre) seedlings. (**A**) Taproot length; (**B**) Total root length; (**C**) Root surface area; (**D**) Root volume; (**E**) Average root diameter. Data are presented as the mean ± SE of six biological replicates. Different lowercase letters above the bars indicate significant differences (*p* < 0.05) between the different root parts and the citrus rootstock species, respectively. CK: control, FeD: iron deficiency, MnD: manganese deficiency, BD: boron deficiency, and ZnD: zinc deficiency.

Further quantitative root morphology analysis results showed that the taproot length was significantly decreased in the Ptr root system under all micronutrient deficiency conditions, while significantly decreased taproot length only occurred under the FeD and MnD treatments in Cre. The total root length, root surface area, and root volume exhibited similar varying tendencies under all micronutrient deficiency conditions in Ptr and Cre roots. In both Ptr and Cre roots, the total root length, root surface area, and root volume decreased significantly under all micronutrient deficiency conditions, except for the Ptr root surface under ZnD conditions. Under MnD conditions, the total root length, root surface area, and root volume of Ptr were significantly higher than the corresponding values in the FeD and BD treatments, while the same was not found for Cre roots. Compared with the control (CK), the average root diameter was significantly increased in the roots of Ptr under FeD and BD conditions but not for the roots of Cre. It is worth noting that the average root diameter was significantly higher under BD conditions than under MnD and ZnD conditions (Figure 4). These results demonstrate that the FeD and BD treatments inhibited all root morphology traits in both Ptr and Cre, except for the average root diameter. In addition, the influence of the MnD and ZnD treatments on root morphology was less than that of the FeD and BD treatments.

**Figure 5:**
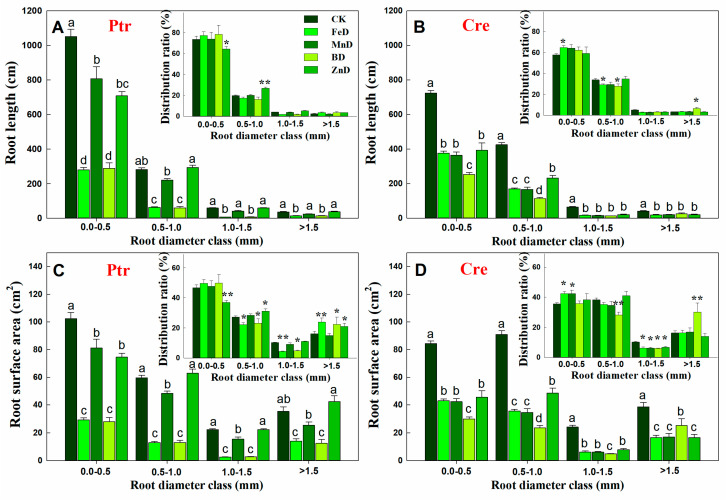
Effects of micronutrient deficiency on the distribution and ratio of root length and root surface area in trifoliate orange (Ptr, **A**,**C**) and red tangerine (Cre, **B**,**D**) seedlings. Data are presented as mean ± SE of six biological replicates. Different lowercase letters above the bars indicate significant differences (*p* < 0.05) between the different root parts and the citrus rootstock species, respectively. Significance of analysis of variance (ANOVA): * *p* < 0.05; ** *p* < 0.01. CK: control, FeD: iron deficiency, MnD: manganese deficiency, BD: boron deficiency, and ZnD: zinc deficiency.

**Figure 6:**
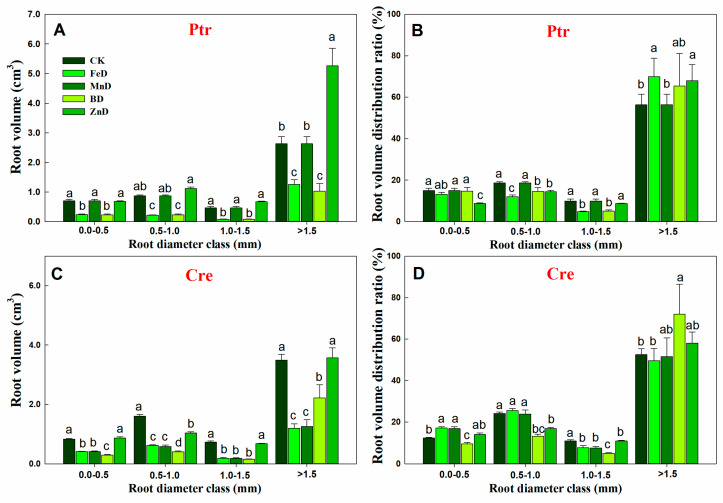
Effects of micronutrient deficiency on the distribution and ratio of root volume in trifoliate orange (Ptr) and red tangerine (Cre) seedlings. (**A**,**B**) Root volume distribution and their ratio of Ptr; (**C**,**D**) Root volume distribution and their ratio of Cre. Data are presented as mean ± SE of six biological replicates. Different lowercase letters above the bars indicate significant differences (*p* < 0.05) between the different root parts and the citrus rootstock species, respectively. CK: control, FeD: iron deficiency, MnD: manganese deficiency, BD: boron deficiency, and ZnD: zinc deficiency.

Compared with the CK treatment, the root length of Ptr was significantly decreased under FeD and BD conditions at all root diameter classes, but for the MnD and ZnD treatments, the root length only decreased significantly when the root diameter was 0.0–0.5 mm. The distribution ratio of Ptr root length only decreased significantly at a root diameter of 0.0–0.5 mm and increased significantly at a root diameter of 0.5–1.0 mm under the ZnD treatment (Figure 5A). Different from Ptr, the root length of Cre was significantly decreased compared with CK under all micronutrient deficiency conditions and in all root diameter classes. The distribution ratio of Cre root length was significantly increased at a root diameter of 0.0–0.5 mm and significantly decreased at a root diameter of 0.5–0.1 mm under the FeD treatment. In contrast, under the BD treatment, the distribution ratio of Cre root length was significantly decreased at a root diameter of 0.5–0.1 mm and significantly increased at a root diameter of >1.5 mm (Figure 5B). The root surface area in Ptr and Cre was significantly inhibited by FeD, MnD, and BD stress in all root diameter classes, but the Ptr root surface area was not significantly reduced under ZnD conditions when the root diameter was >0.5 mm. The distribution ratio of the Ptr root surface area was significantly decreased at a root diameter of 0.0–0.5 mm but increased at root diameters of 0.5–1.0 mm and >1.5 mm under the ZnD treatment. For the FeD and BD treatments, the distribution ratio of the Ptr root surface area decreased significantly at root diameters of 0.5–1.0 and 1.0–1.5 mm but increased at a root diameter of >1.5 mm. Different from Ptr, the distribution ratio of the Cre root surface area was significantly increased at a root diameter of 0.0–0.5 mm and significantly decreased at a root diameter of 1.0–1.5 mm under the FeD and MnD treatments. For BD treatments, the distribution ratio of the Cre root surface area was decreased significantly at root diameters of 0.5–1.0 and 1.0–1.5 mm but increased at a root diameter of >1.5 mm (Figure 5C,D). The effects of micronutrient deficiency on the distribution and ratio of root volume in Ptr and Cre seedlings were also analyzed in this work (Figure 6). Compared with the CK treatment, the root volume of Ptr was decreased significantly under FeD and BD conditions at all root diameter classes, but there was no significant difference for the MnD and ZnD treatments. The root volume only increased significantly when the root diameter was >1.5 mm under the ZnD treatment. For Cre, the root volumes decreased significantly under FeD, MnD, and BD conditions at all root diameter classes, but the root volume only decreased significantly when the root diameter was 0.5–1.0 mm under the ZnD treatment. The distribution ratio of Ptr root volume decreased significantly at a root diameter of 0.5–1.5 mm and increased significantly at a root diameter of >1.5 mm under the FeD and BD treatment. But, no significant change was found in Ptr root volume under MnD treatment. For Cre, the distribution ratio of root volume decreased significantly at a root diameter of 0.0–1.5 mm and increased significantly at a root diameter of >1.5 mm under the BD treatment. The distribution ratios of root volume were significantly increased at a root diameter of 0.0–1.0 mm but decreased at root diameters of 1.0–1.5 mm under the FeD and MnD treatment and only decreased at root diameters of 0.5–1.5 mm for ZnD treatment.

### 2.5. Mineral Nutrient Concentration Analysis

**Figure 7:**
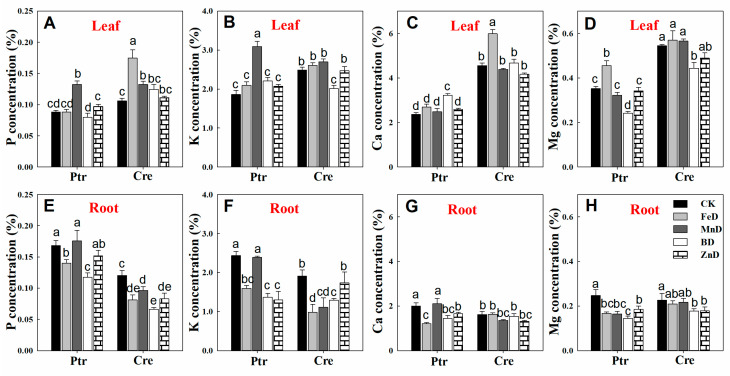
Effects of micronutrient deficiency on macronutrient concentrations (%) in the leaves and roots of trifoliate orange and red tangerine seedlings. P concentration in leaf (**A**) and root (**E**); K concentration in leaf (**B**) and root (**F**); Ca concentration in leaf (**C**) and root (**G**); Mg concentration in leaf (**D**) and root (**H**).Trifoliate orange (Ptr) and red tangerine (Cre) seedlings were grown under different micronutrient deficiency conditions for 12 weeks. Data are presented as means ± SE of nine replicates (n = 9, one plant for each replicate). Different lowercase letters above the bars indicate significant differences (*p* < 0.05) between different growth conditions. CK: control, FeD: iron deficiency, MnD: manganese deficiency, BD: boron deficiency, and ZnD: zinc deficiency.

The concentrations of mineral macronutrients (P, K, Ca, and Mg) in the leaves and roots of Ptr and Cre seedlings are presented in Figure 7. MnD significantly increased the P concentrations in the leaves of Ptr and Cre, and a similar tendency was found in the leaves of Cre under FeD conditions. In contrast, the root P concentration was significantly reduced in both Ptr and Cre, except for in MnD- and ZnD-treated Ptr. For K, the MnD treatment significantly enhanced the leaf K concentration in Ptr, while the BD treatment significantly decreased the leaf K concentration in Cre. All treatments reduced the root K concentrations, except for MnD in Ptr and ZnD in Cre. The leaf Ca concentration was significantly elevated in Ptr under BD and in Cre under FeD, whereas no significant changes were detected in root Ca concentrations, except for in FeD-, BD-, and ZnD-treated Ptr seedlings. The Mg concentration in the leaves of Ptr and Cre was reduced under BD conditions, whereas it was increased by the FeD treatment. The root Mg concentration was also reduced under micronutrient deficiency conditions, except for in FeD- and MnD-treated Cre seedlings.

**Figure 8:**
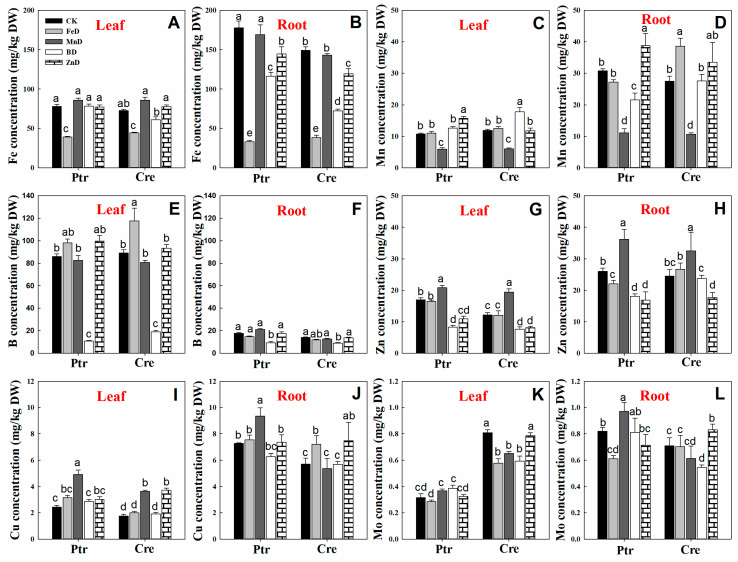
Effects of micronutrient deficiency on the micronutrient concentrations (mg/kg DW) in the leaves and roots of trifoliate orange and red tangerine seedlings. Fe concentration in leaf (**A**) and root (**B**); Mn concentration in leaf (**C**) and root (**D**); B concentration in leaf (**E**) and root (**F**); Zn concentration in leaf (**G**) and root (**H**); Cu concentration in leaf (**I**) and root (**J**); Mo concentration in leaf (**K**) and root (**L**). Trifoliate orange (Ptr) and red tangerine (Cre) seedlings were grown under different micronutrient deficiency conditions for 12 weeks. Data are presented as means ± SE of nine replicates (n = 9, one plant for each replicate). Different lowercase letters above the bars indicate significant differences (*p* < 0.05) between different growth conditions. CK: control, FeD: iron deficiency, MnD: manganese deficiency, BD: boron deficiency, and ZnD: zinc deficiency.

**Figure 9:**
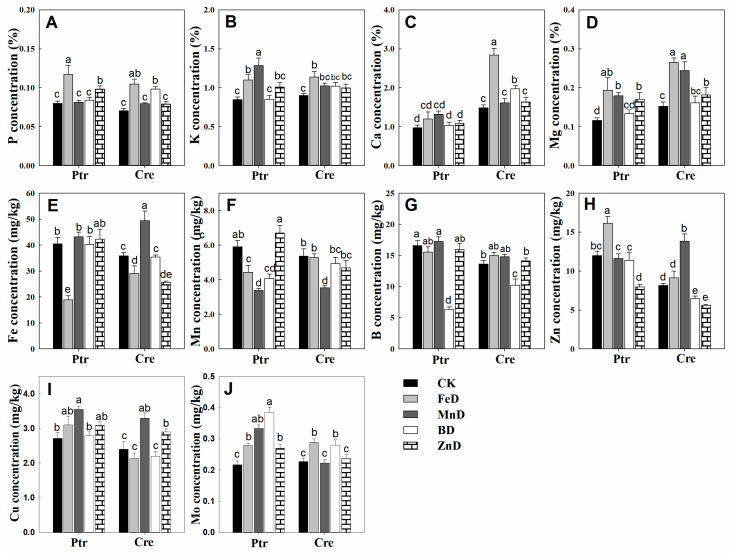
Effects of micronutrient deficiency on the mineral nutrient concentrations in the stems of trifoliate orange and red tangerine seedlings. (**A**) P concentration; (**B**) K concentration; (**C**) Ca concentration; (**D**) Mg concentration; (**E**) Fe concentration; (**F**) Mn concentration; (**G**) B concentration; (**H**) Zn concentration; (**I**) Cu concentration; (**J**) Mo concentration. Trifoliate orange (Ptr) and red tangerine (Cre) seedlings were grown under different micronutrient deficiency conditions for 12 weeks. Data are presented as means ± SE of nine replicates (n = 9, one plant for each replicate). Different lowercase letters above the bars indicate significant differences (*p* < 0.05) between different growth conditions. CK: control, FeD: iron deficiency, MnD: manganese deficiency, BD: boron deficiency, and ZnD: zinc deficiency.

The concentrations of micronutrients (Fe, Mn, B, Zn, Cu, and Mo) in the leaves and roots of Ptr and Cre seedlings are presented in Figure 8. The Fe concentrations were significantly higher in the roots than in the leaves of Ptr and Cre seedlings, and the Fe concentrations were significantly reduced under FeD conditions in both the leaves and roots. The root Fe concentrations were also significantly reduced under BD and ZnD conditions (Figure 8A,B). The Mn concentrations declined under MnD conditions in both the leaves and roots of Ptr and Cre seedlings, and the Mn concentrations were also decreased in BD-treated Ptr. In contrast, the Mn concentrations were increased in the leaves and roots of Ptr under ZnD conditions (Figure 8C,D). The B concentrations were significantly higher in the leaves than in the roots of Ptr and Cre seedlings under CK conditions. The BD treatment significantly reduced the B concentrations in the leaves and roots of both Ptr and Cre seedlings, while the B concentration increased in the MnD-treated leaves of Cre seedlings (Figure 8E,F). The leaf concentrations of Zn were significantly reduced in both ZnD- and BD-treated Ptr and Cre seedlings, and the same trend was found in roots, except for in BD-treated Cre seedlings. Interestingly, elevated Zn concentrations were detected in the leaves and roots of both rootstock species under MnD treatment conditions (Figure 8G,H). In the case of Cu, the Cu concentration increased significantly under MnD treatment in both Ptr leaves and roots, and the same trend was also found in MnD- and ZnD-treated Cre seedlings (Figure 8I,J). The Mo concentration showed no significant change in the leaves of Ptr under micronutrient deficiency conditions, whereas the Mo concentration decreased significantly in Cre, except for under the ZnD treatment. In the roots, the Mo concentrations were reduced in FeD-treated Ptr and BD-treated Cre, whereas they were increased in MnD-treated Ptr and ZnD-treated Cre (Figure 8K,L). The concentrations of mineral nutrients in the stems of Ptr and Cre seedlings were also determined (Figure 9). There was no significant decrease in the concentrations of macronutrients (P, K, Ca, and Mg) in the stem of Ptr and Cre seedlings under micronutrient deficiency conditions. Especially in the treatment of FeD, the concentrations of macronutrients in stems increased significantly in both Ptr and Cre, except Ca in Ptr seedlings. The concentrations of Fe, Mn, B, and Zn in stems of Ptr and Cre decreased significantly in the corresponding treatments of FeD, MnD, BD, and ZnD. There was no significant decrease in the Cu and Mo concentrations in the stem of Ptr and Cre seedlings under micronutrient deficiency conditions. Moreover, MnD promoted the increase in stem Cu concentration, and FeD and BD promoted the increase in stem Mo concentration in both Ptr and Cre.

### 2.6. Dynamic Analysis of Lateral Roots Development

A short-term experiment was conducted to investigate the effects of micronutrient deficiency on LR formation and growth. As shown in Table 2, the Ptr first LR number was significantly lower under the FeD and MnD treatments than under the CK treatment, although not under the BD and ZnD treatments. In contrast, there was no significant decline in the number of first LRs in Cre, except for under FeD stress. The lengths of all first and second LRs were inhibited under micronutrient deficiency conditions, except for in ZnD-treated Ptr seedlings. Under FeD and MnD conditions, the number of second LRs decreased significantly in both rootstock species. Under ZnD conditions, the number of second LRs only declined in Cre. Interestingly, no significant changes occurred in the root number in all cases (two species and two kinds of LRs) under BD stress. Therefore, the root density of second LRs increased under BD stress. In contrast, this density was inhibited under MnD and ZnD conditions only in Cre seedlings.

**Figure 10:**
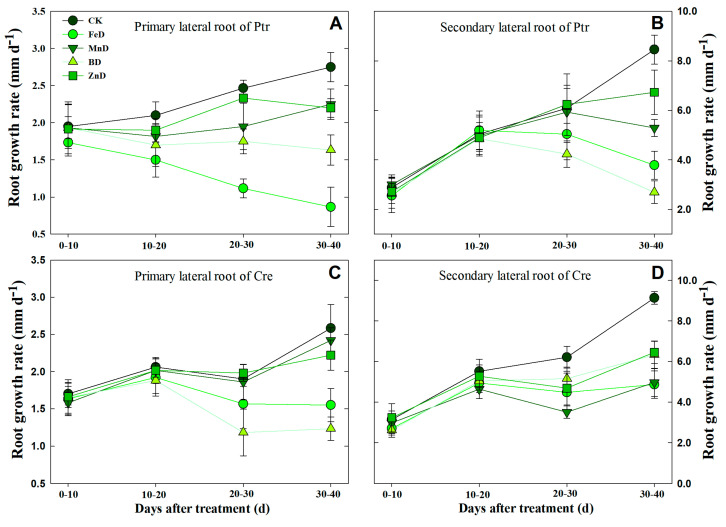
Dynamic analysis of the lateral root growth rates of trifoliate orange (Ptr) and red tangerine (Cre) seedlings under micronutrient deficiency conditions. (**A**) The primary lateral root growth rate of Ptr; (**B**) The secondary lateral root growth rate of Ptr; (**C**) The primary lateral root growth rate of Cre; (**D**) The secondary lateral root growth rate of Cre. Trifoliate orange and red tangerine seedlings were grown under different micronutrient deficiency conditions for 40 days. CK: control, FeD: iron deficiency, MnD: manganese deficiency, BD: boron deficiency, and ZnD: zinc deficiency. All results regarding the per-plant root growth rate data are the average value (±SD) from nine seedlings.

Based on the above results, dynamic analyses of LR development were conducted in Ptr and Cre under micronutrient deficiency conditions by determining the different-order LR growth rates (Figure 10). Under normal (CK) conditions, the growth rates of first and second LRs in both rootstock species displayed changes with treatment time. Under FeD conditions, the Ptr root elongation rate decreased significantly in first LRs, but it increased at first and then decreased in second LRs. For Cre, the rate remained unchanged after 10 days of FeD treatment in both LRs. Under MnD and ZnD conditions, the root growth rate did not change significantly in the first LR of Cre, but the root growth rate dropped significantly at the end of the treatments in the second LR of Cre and in both the first and second LRs of Ptr. For BD-treated Ptr, the root growth rate remained unchanged during the entire treatment period in the first LR, whereas the root growth rate first increased and then declined in the second LR. However, for BD-treated Cre, the root growth rate showed a downward trend in the first LR and a slowly increasing trend in the second LR during the treatment time (Figure 10).

## 3. Discussion

### 3.1. Mineral Nutrient Distribution in Citrus Root

The present work analyzed the mineral nutrient concentrations in taproots and lateral roots to clarify the distribution of mineral nutrients in different parts of the citrus root system (Figure 1 and Figure 2). The results suggested that there were obvious differences in the mineral nutrient distribution characteristics of root partitioning. Furthermore, the results revealed that the content of mineral nutrients in different parts of the roots varied and that the distribution of the same mineral nutrients in different parts of the roots was also different. This conclusion is similar to the findings of previous research on citrus leaves [25]. Investigating the spatial distribution characteristics of mineral nutrients in citrus roots can not only serve as a reference for effective nutrient management in citrus production but also shed light on the formation mechanism of root mineral nutrient deficiency symptoms, providing a theoretical basis for nutritional diagnosis in citrus.

The spatial distribution of mineral nutrients in different leaf parts is well known in higher plants, including citrus plants, based on research conducted using elemental imaging or ICP-MS [25,26,27]. However, very few studies have been performed on plant roots, and the spatial distribution of mineral nutrients in different root parts of citrus plants is unclear. As shown in Figure 1, there were significant differences in the spatial distribution of mineral nutrients in different root parts in both Ptr and Cre. The mechanism underlying these differences requires further study. There are two main sources of mineral nutrients in citrus roots: mineral nutrients absorbed by the roots themselves and mineral nutrients transported from elsewhere in the plant. It is well known that the younger the root system, the better its ability to absorb and preferentially use mineral nutrients. Previous research demonstrated that the P and K contents of roots were unaffected by P distribution patterns, whereas the Ca and Mg contents were markedly increased in older tissues when P was present in the ambient solution [28]. These data support the concept that internally translocated P and externally absorbed P have differing effects at a given root site because the former does not reach the site in the same quantity as the latter. In this study, except for the P concentrations in Ptr, the last two levels of LRs (second and third LRs) exhibited the highest macronutrient concentrations (Figure 1). In addition, the differential distribution of mineral nutrients may be influenced by variations in transpiration, differences in the root absorption of mineral elements, and the mobility of elements.

Interestingly, based on the results of PCA, B and P were very close in terms of their spatial distribution (Figure 2B). This result implies that P and B may play similar roles in root growth and development. The P nutrition status exerts very fine adjustment actions on plant root system growth. The soil P status induces or inhibits plant taproot or LR formation and development, thereby altering the root morphology and architecture [1,29,30,31]. Our previous studies showed that the P concentrations in the roots of seven types of citrus rootstock seedlings declined significantly under BD conditions [22]. This decrease in P associated with BD conditions may explain why the growth and development of citrus rootstock roots were significantly inhibited under BD. Nutrient interaction is an important factor affecting the efficient use of nutrients and yield formation of crops, and the results of this study suggest that the interaction relationship between B and P in citrus necessitates further study. In addition, there were no elements distributed in the negative area of PC1 (Figure 2B). This suggests that root mineral nutrients are mainly derived from uptake by roots rather than translocation from other parts of the plant. As shown in Figure 2B, Mo, Mn, Cu, and Fe contributed significantly to PC1, while B, Cu, Mo, and Zn contributed significantly to PC2 in both Ptr and Cre. Many previous studies have also demonstrated that micronutrients play important roles in the growth and development of plant roots. For example, under FeD conditions, the number of root tips and the length of the main root increased significantly, whereas the activity and the number of roots decreased significantly in young seedlings of Malus hupehensis [32]. In addition, compared with the Zn-rich conditions, low-Zn conditions reduced the taproot length per plant of ‘Newhall’ citrus by 19.30% and that of ‘Ponkan’ citrus by 20.07% [33]. Altogether, these results indicate that micronutrients play essential roles in citrus root growth and development.

### 3.2. Adaptive Growth of Root and Stem Under Micronutrient Deficiency Conditions

The results of the current study indicated that the growth and development of root and stem were significantly inhibited under micronutrient deficiency conditions (Figure 3, Figure 4, Figure 5 and Figure 6 and Table 1). Previous studies have demonstrated that B deficiency treatment significantly decreased the stem length (seedling height) of 7 different citrus rootstock seedlings, including Ptr and Cre [22]. The stem morphologies were changed significantly after 100 days of iron deficiency, but there were no significant differences found in stem length and stem dry weight among Zhique (tolerant to iron deficiency), Xiangcheng (tolerant to iron deficiency), and trifoliate orange (sensitive to iron deficiency) [16]. Our results were consistent with those of previous studies (Table 1). However, stem growth and development did not show characteristics of adaptation to micronutrient deficiency stress. Compared with the stem, the growth and development of the root showed a strong adaptability to micronutrient deficiency stress. Under plant nutrient deficiency, the development of plant roots, root morphology, and architecture undergo a series of proactive changes to adapt to the stress of nutrient deficiency and maintain the normal growth of plants, and the same is true for micronutrient deficiency. To mitigate P scarcity, plants express an array of morphological, physiological, and metabolic changes known as the phosphate starvation response [30,34,35,36]. Previous studies have shown that short-term low P stress can promote the elongation of LRs and increase the number of LRs in tobacco. The increased LR length and number can alleviate the decreased P content in each part of the plant under P deficiency stress and may be useful in breeding tobacco varieties with stronger root systems [29]. The same conclusion was also found in a previous study on P deficiency in apples [34]. In the present study, a short-term experiment was conducted to investigate the effects of micronutrient deficiency on LR formation in Ptr seedlings. As shown in Table 2, there were no significant decreases in the numbers of first and second LRs in Ptr seedlings under BD and ZnD conditions. Dynamic analyses of LR development were conducted in Ptr and Cre under micronutrient deficiency conditions, and the results indicated that the growth rates of first and second LRs did not decrease significantly compared with the CK under short-term (10 days) B deficiency stress (Figure 10). However, our previous research demonstrated that the LR number and length in Ptr were significantly reduced under long-term (8 or 10 weeks) B deficiency stress [22]. In addition, plants can respond to nutrient deficiency stress by regulating phytohormone levels, but long-term nutrient deficiency stress can lead to root tip necrosis and, therefore, a decrease in the number of root tips, thus affecting the synthesis and transport of cytokines and auxin. Therefore, the root number, root length, and growth rate of LRs of citrus at all levels (first and second LRs) increased significantly under short-term micronutrient deficiency stress to adapt to the nutrient-limited environment, but this adaptability would be weakened under long-term stress. Overall, citrus alters its root morphology and biological traits as a nutrient acquisition strategy to maintain maximal micronutrient acquisition and growth.

Based on previous studies, field rootstock root management was closely related to micronutrient fertilization. But, the growers did not pay much attention to micronutrient fertilizer application in citrus field production. A recent study suggested that the number of root tips and the total root surface area were important parameters of root morphology in relation to the drought tolerance of plants. They could be the target traits for breeding and selection of rootstocks for drought tolerance. In addition, the trifoliate orange hybrid ZZ-022 demonstrated good potential as a superior rootstock due to its good root system and drought tolerance [37]. Selecting the right rootstock is the best way to obtain excellent root morphology, as different rootstocks have different types of root morphology. Ptr, which had the lowest canopy volume and the highest yield efficiency, presented the highest total soluble solids. The results indicated that Ptr would be the best rootstock for navel oranges in terms of fruit quality [38]. Similar studies have also been conducted on the effects of different rootstocks on ‘Ehime 28’ orange vegetative organs and the effects of approach-grafting rootstock combinations on the fruit quality of Lane Late navel orange [39,40]. The results of our current study provided a theoretical basis for the selection of rootstock in citrus production.

### 3.3. Micronutrient Deficiency Affects the Formation and Growth of LR

Several studies have demonstrated that micronutrients are involved in the growth and development of plant roots [1,3,33], with micronutrient deficiency or excess altering root morphology and having a pronounced effect on the formation and growth of LRs [8,41]. The LR formation or emergence mechanisms are well known under several macronutrient-limited conditions, such as P, K, and S deficiency [11], but are less well studied under micronutrient-limited conditions.

In citrus, B is one of the most extensively studied micronutrients and has the most significant effect on root growth and development, morphology, and architecture. Previous studies on citrus rootstock roots have revealed that the LR number increases significantly under B deficiency conditions [20,22]. For example, B deficiency significantly inhibited the growth of Poncirus trifoliata seedlings, significantly decreased the mean LR length and root number, and significantly enhanced the root diameter and LR primordial density after 30 days of treatment; furthermore, under 90 days of B deficiency treatment, the root number, length, and surface area were significantly reduced in fragrant citrus and trifoliate orange [20]. These results were consistent with the present findings. In the current study, a short-term experiment was conducted to investigate the effects of BD on LR formation in Ptr and Cre seedlings (Table 2). However, the formation mechanism of citrus LRs under B starvation conditions remains unclear. Therefore, dynamic analyses of LR development were conducted in Ptr and Cre under BD conditions by determining the different-order LR growth rates (Figure 10). The results further clarified the dynamic effects of BD on citrus LR growth.

For the deficiency of other micronutrients, few studies focusing solely on LR growth and development have been reported in citrus. In this work, FeD had the most significant effect on the growth and development of citrus roots among all micronutrient deficiencies in both Ptr and Cre seedlings (Figure 3, Figure 4, Figure 5 and Figure 10). This may be due to the significantly higher concentration of Fe in plants than other micronutrients. In addition, the Fe concentration was significantly higher in citrus roots than in stems and leaves (Figure 8). The Fe concentration was remarkably higher in both the second LR and third LR than in other parts of the root of Ptr. This trend was also observed in Cre. The Fe concentration was significantly higher in the first LR than in the root collar for both rootstocks (Figure 1). Our previous work showed that, under normal conditions, the Fe concentrations in Ptr and Cre roots were 10.95 and 14.11 times that in the leaves, respectively [22]. Recent research has demonstrated that the LR number is significantly reduced under FeD conditions in trifoliate orange [16], and these results confirm that FeD can significantly affect the growth, development, and morphology of citrus roots.

As mentioned previously, extensive research has been conducted on citrus roots. However, the mechanism through which micronutrient deficiency affects citrus LR development remains unclear. LR formation is a multistep developmental process in which auxin and peptide hormones play essential roles [42]. Recent studies in Arabidopsis have revealed that the mitogen-activated protein kinase (MAPK) cascade MKK4/MKK5–MPK3/MPK6 functions in both a noncanonical auxin signaling pathway and the IDA peptide signaling pathway to regulate LR morphogenesis and emergence, respectively [43,44]. Therefore, investigating the effects of micronutrient deficiency on the growth, development, and morphology of citrus LRs from the perspective of auxin regulation is a promising avenue for future research.

## 4. Materials and Methods

### 4.1. Plant Materials and Treatment

Two citrus rootstocks, trifoliate orange (*Poncirus trifoliata* L. Raf.) and red tangerine (*Citrus reticulata* Blanco), were employed in the present study. The seeds of these two rootstocks were germinated, and seedling culture proceeded following a previously described method [45]. For the determination of the root system mineral nutrient distribution in different root parts (the root collar, taproot, and three levels of lateral roots), the seedlings were cultured in 15 L black plastic pots (with 30 cm height and 25 cm internal diameter) containing soil for 6 months. The soil characteristics were as follows: organic matter 19.91 ± 0.49 g/kg, total N 0.97 ± 0.12 g/kg, available P 21.14 ± 0.64 mg/kg, available K 115.25 ± 11.36 mg/kg, and pH 5.92 ± 0.52. For the micronutrient (Fe, Mn, B, and Zn) deficiency experiment, 4-week-old seedlings of similar taproot length were selected and then transferred to 5 L black pots containing nutrient-free medium composed of quartz sand–perlite (1:1, *v*/*v*) according to a published method [46,47]. Plants were cultured in a growth chamber with normal culture parameters according to the method of Zhou et al. [22]. Plants were irrigated twice a week with modified Hoagland’s No. 2 nutrient solution containing 6 mM KNO_3_, 4 mM Ca(NO_3_)_2_, 1 mM NH_4_H_2_PO_4_, 2 mM MgSO_4_, 9 µM MnCl_2_, 0.8 µM ZnSO_4_, 20 µM H_3_BO_3_, 0.3 µM CuSO_4_, 0.01 µM H_2_MoO_4_, and 50 µM Fe-EDTA [45]. Irrigating with this control nutrient solution (CK), seedlings were precultured for 2–3 weeks until new white roots appeared. Then, seedlings were irrigated with 0 µM Fe-EDTA nutrient solution for the Fe deficiency (FeD) treatment, 0 µM MnCl_2_ nutrient solution for the Mn deficiency (MnD) treatment, 0 µM H_3_BO_3_ nutrient solution for the B deficiency (BD) treatment, and 0 µM ZnSO_4_ nutrient solution for the Zn deficiency (ZnD) treatment. The treatments began on 17 April 2023 and concluded on 4 July 2023 (12 weeks), when visible symptoms had appeared. For the dynamic analysis of LR development, a short-term (40-day) experiment was conducted to investigate the effects of micronutrient deficiency on the LR formation and growth rate in Ptr and Cre seedlings. In this experiment, the plant material culture and micronutrient deficiency treatments were the same as described previously.

### 4.2. Sampling and Measurement of Plant Growth Parameters

At the end of the experiment, nine plants per treatment were harvested randomly, rinsed in deionized water, and carefully blotted with tissue paper. Then, the materials were divided into leaf, stem, and root tissues. The fresh materials were placed into a forced air oven at 105 °C for 15 min and then heated at 75 °C until constant weights were reached to determine their stem and root dry weights (g). All dried samples were then ground into fine powder to determine the mineral nutrient concentrations in tissues. The stem length (cm) and taproot length (cm) were measured using a scaled ruler. Stem diameter (mm) was measured using a vernier caliper.

### 4.3. Root Morphology and Analysis

Nine seedlings were randomly sampled from each treatment group and rinsed with deionized water. For root morphology analysis, the root samples were scanned using an Epson digital scanner Expression 10000XL 1.0 (Epson Inc., Suwa City, Nagano Prefecture, Japan), and the image was analyzed using WinRhizo Pro (S) v. 2009c software (Regent Instruments Inc., Québec, QC, Canada). The root traits examined in this study included the total root length, root surface area, root volume, and root number. The root length and root surface area within a certain root diameter (0.0–0.5 mm, 0.5–1.0 mm, 1.0–1.5 mm, and >1.5 mm) were also analyzed using this software.

### 4.4. Dynamic Analysis of LR Development

A short-term (40-day) experiment was conducted to investigate the effects of micronutrient deficiency on the LR formation and growth rate in Ptr and Cre seedlings. After 40 days of experimental treatment, the 1st LR and 2nd LR were separated. The root number and length of the 1st and 2nd LRs were scanned with an Epson digital scanner, and the image was analyzed using WinRhizo Pro (S) v. 2009c software. The dynamic analysis of LR development in Ptr and Cre was conducted under micronutrient deficiency conditions by determining the growth rates of different LRs. The lengths of 1st and 2nd LRs were measured every 10 days until day 40, and the LR growth rates were analyzed.

### 4.5. Determination of Mineral Nutrients

The mineral nutrient concentrations of P, K, Ca, Mg, Fe, Mn, Zn, B, Cu, and Mo in different plant tissues were determined following the method described by Storey and Treeby [48]. In brief, 0.50 g of each sample was dry-ashed in a muffle furnace at 500 °C for 6 h, followed by dissolution in 0.1 N HCl. The mineral nutrient concentrations were then determined using ICP-MS 7900 (Agilent Technologies Inc., Santa Clara, CA, USA).

### 4.6. Experimental Design and Statistical Analysis

The experiment was set up in a completely randomized 5 × 2 factorial design with one control (CK) and four micronutrient deficiency solutions (FeD, MnD, BD, and ZnD) combined with two citrus rootstocks. The values are presented as means ± SE of nine seedlings. The data underwent analysis of variance (ANOVA) in SAS 8.1 software (SAS Institute Inc., Cary, NC, USA), and the differences were compared using Duncan’s test with a significance level of *p* < 0.05. PCA was applied to analyze the dimensionality reduction of the ion group of the root based on the content correlation matrix of 10 elements in the root. The first two principal components were extracted to design the score diagram and the load diagram.

## 5. Conclusions

This study demonstrated that citrus root growth and development, morphology, and architecture were affected by micronutrients, as well as the morphology of the stem. In the present work, the distribution of mineral nutrients in different parts of the citrus root system suggested that there were obvious differences in the element distribution characteristics under root partitioning and that the element concentrations and ion composition in the second LR and third LR were very different compared with other root parts. The analysis of root morphology under micronutrient deficiency demonstrated that the root growth was significantly inhibited in both Ptr and Cre under FeD and BD conditions compared with the MnD and ZnD treatments, but Cre roots exhibited better performance than Ptr roots. The nutrient concentrations in Ptr and Cre seedlings under micronutrient deficiency revealed that single micronutrient deficiencies affected both their own concentrations and the concentrations of other mineral nutrients, whether in the roots or in stems and leaves. The dynamic analysis of LR development revealed that the growth rates of the first LR and second LR did not decrease significantly compared with CK under short-term micronutrient deficiency stress. To summarize, these results suggest that micronutrients play important roles in citrus root growth and development as well as RSA modulations. The present work on the spatial distribution characteristics and micronutrient deficiency of citrus roots provides a theoretical basis for effective micronutrient fertilization and the diagnosis of micronutrient deficiency symptoms in citrus.

## Figures and Tables

**Table 1:** Effects of micronutrient deficiency stress on stem and root growth parameters in trifoliate orange and red tangerine seedlings.

Species	Growth Parameters	CK	FeD	MnD	BD	ZnD
Ptr	Stem length (cm)	83.64 ± 3.37 b	29.21 ± 3.50 d	34.60 ± 6.35 d	74.50 ± 6.37 c	95.98 ± 4.47 a
	Stem diameter (mm)	4.57 ± 0.08 b	4.56 ± 0.12 b	3.12 ± 0.23 c	5.94 ± 0.11 a	5.13 ± 0.14 ab
	Stem dry weight (g)	2.39 ± 0.25 b	0.89 ± 0.10 c	0.50 ± 0.15 c	3.18 ± 0.50 a	3.32 ± 0.21 a
	Root dry weight (g)	1.58 ± 0.21 a	0.34 ± 0.10 c	1.07 ± 0.09 b	0.58 ± 0.04 c	1.40 ± 0.18 a
Cre	Stem length (cm)	76.92 ± 2.60 a	27.76 ± 2.08 c	50.32 ± 5.13 b	30.62 ± 5.38 c	30.54 ± 5.98 c
	Stem diameter (mm)	4.81 ± 0.17 a	3.26 ± 0.07 b	3.41 ± 0.21 b	4.15 ± 0.33 ab	3.46 ± 0.20 b
	Stem dry weight (g)	2.49 ± 0.20 a	0.52 ± 0.04 b	0.97 ± 0.16 b	0.87 ± 0.28 b	0.87 ± 0.20 b
	Root dry weight (g)	1.39 ± 0.06 a	0.57 ± 0.02 c	0.90 ± 0.08 b	0.52 ± 0.05 c	0.60 ± 0.07 c

Note: Trifoliate orange (Ptr) and red tangerine (Cre) seedlings were grown under different micronutrient deficiency conditions for 12 weeks. CK: control, FeD: iron deficiency, MnD: manganese deficiency, BD: boron deficiency, and ZnD: zinc deficiency. Different lowercase letters following the mean values indicate significant differences (*p* < 0.05) between different growth conditions.

**Table 2:** Effects of micronutrient deficiency on lateral root formation and growth in trifoliate orange and red tangerine seedlings.

Species	Treatment	Primary Lateral Roots		Secondary Lateral Roots		
		Number	Length (cm)	Number	Length (cm)	Density (Number/cm)
Ptr	CK	19.56 ± 1.17 a	8.23 ± 0.55 a	24.44 ± 2.30 a	2.90 ± 0.08 a	2.96 ± 0.21 b
	FeD	13.22 ± 1.11 c	4.85. ± 0.31 c	14.33 ± 0.74 c	1.92 ± 0.47 c	3.02 ± 0.32 b
	MnD	16.78 ± 1.29 b	7.07 ± 0.63 b	19.44 ± 2.85 b	2.48 ± 0.23 b	2.90 ± 0.38 b
	BD	20.67 ± 1.61 a	4.93 ± 0.34 d	23.89 ± 2.68 a	1.15 ± 0.13 d	4.93 ± 0.61 a
	ZnD	18.11 ± 2.42 ab	7.66 ± 0.60 ab	22.89 ± 4.74 ab	2.62 ± 0.76 ab	3.14 ± 0.48 b
Cre	CK	22.33 ± 1.61 a	7.67 ± 0.74 a	19.83 ± 1.74 a	2.66 ± 0.13 a	2.67 ± 0.26 b
	FeD	16.00 ± 1.18 b	6.18 ± 0.33 cd	15.83 ± 0.95 b	2.05 ± 0.14 b	2.57 ± 0.09 b
	MnD	18.17 ± 1.30 ab	6.54 ± 0.44 c	13.83 ± 1.08 c	1.78 ± 0.14 c	2.14 ± 0.17 c
	BD	21.17 ± 1.17 a	5.51 ± 0.57 d	20.67 ± 1.94 a	1.44 ± 0.17 d	3.96 ± 0.53 a
	ZnD	20.33 ± 2.09 a	6.88 ± 0.43 b	13.33 ± 2.54 c	1.79 ± 0.19 c	1.98 ± 0.20 c

Note: Trifoliate orange (Ptr) and red tangerine (Cre) seedlings were grown under different micronutrient deficiency conditions for 40 days. CK: control, FeD: iron deficiency, MnD: manganese deficiency, BD: boron deficiency, and ZnD: zinc deficiency. Different lowercase letters following the mean values indicate significant differences (*p* < 0.05) between different growth conditions.

## Data Availability

Data are contained within the article.
